# Expanding the genetic and clinical characteristics of Protocadherin 19 gene mutations

**DOI:** 10.1186/s12920-022-01313-w

**Published:** 2022-08-17

**Authors:** Giovanni Battista Dell’Isola, Elisabetta Mencaroni, Antonella Fattorusso, Giorgia Tascini, Paolo Prontera, Valentina Imperatore, Giuseppe Di Cara, Pasquale Striano, Alberto Verrotti

**Affiliations:** 1 Pediatric Clinic, Department of Surgical and Biomedical Sciences, University of Perugia, Piazzale Giorgio Menghini 1, Perugia, Italy; 2 Medical Genetics Unit, Hospital Santa Maria della Misericordia, Perugia, Italy; 3 Pediatric Neurology and Muscular Diseases Unit, IRCCS “G. Gaslini” Institute, Genoa, Italy; 4 Department of Neurosciences, Rehabilitation, Ophthalmology, Genetics, Maternal and Child Health, University of Genoa, Genoa, Italy

**Keywords:** PCDH19, AEDs, Epilepsy genetics, Case report

## Abstract

**Background:**

PCDH19-related epilepsy is a rare X-linked type of epilepsy caused by genomic variants of the Protocadherin 19 (*PCDH19*) gene. The clinical characteristics of PCDH19-related epilepsy are epileptic and non-epileptic symptoms with highly variable severity among patients.

**Case presentation:**

We present a case of a 4-year old female with PCDH19-related epilepsycaused by new variants in the *PCDH19* gene. Our patient was admitted for the first time at the age of 12 months for seizure clusters arising under condition of apyrexia. The electroencephalography (EEG) showed frontal paroxysmal activity. The genetic analysis identified the two variants c.1006G > A (p.Val336Met) and c.1014C > A (p.Asp338Glu) in the gene PCDH19. The patient was treated with Carbamazepine and Clonazepam achieving the disappearance of seizures. During the follow-up, the neurological examination was persistently normal with neither cognitive impairment nor behavior disturbances. From 2 years of age EEG controls were persistently normal.

**Conclusion:**

This patient presents two novel variants of the *PCDH19* gene associated with a mild form of epilepsy with normal cognitive development with an apparently better prognosis. According to our experience, the dual therapy with Carbamazepine and Clonazepam has led to a good control of seizures.

## Background

The Protocadherin 19 (*PCDH19*) gene is located on chromosome Xq22.1 and it is involved in neuronal connections and signal transduction [1, 2]. *PCDH19* genomic variants, generally involving the exon1, are correlated with the developmental and epileptic encephalopathy 9 (DEE9) [3]. The phenotypic expression seems to be associated with random X inactivation in heterozygosity. Indeed, the coexistence of mutated cells with wild type cells prevents the construction of the normal neuronal network, therefore carrier males are unaffected [4]. The clinical characteristics of PCDH19-related epilepsy are represented by early onset (6–36 months) seizures, generally sensitive to fever [5]. Seizure types are highly variable from mild epilepsy to epileptic encephalopathy, overlapping with epileptic syndromes including febrile seizure plus (FS +) [6]. FS + is a neonatal/infantile epilepsy syndrome characterized by febrile seizures extended beyond the typical age where these are expected to resolve and/or be accompanied by afebrile seizures [7]. Non-epileptic features include intellectual disability and behavior disturbances occurring in 75.4% and 55.4% of patients respectively [8]. Seizures become less severe with adolescence while cognitive retardation and behavior disorder represent the main disabilities of adult patients with *PCDH19* genomic variants [8]. Brain magnetic resonance imaging (MRI) is generally normal and there are no peculiar electroencephalographic (EEG) features of *PCDH19* mutation. Almost 150 *PCDH19* genomic variants have been described as either familiar clustering or de novo [9].

Here, we report a case of a 4-years old female with PCDH19-related epilepsy caused by new variants in the *PCDH19* gene.

## Case presentation

**Fig. 1:**
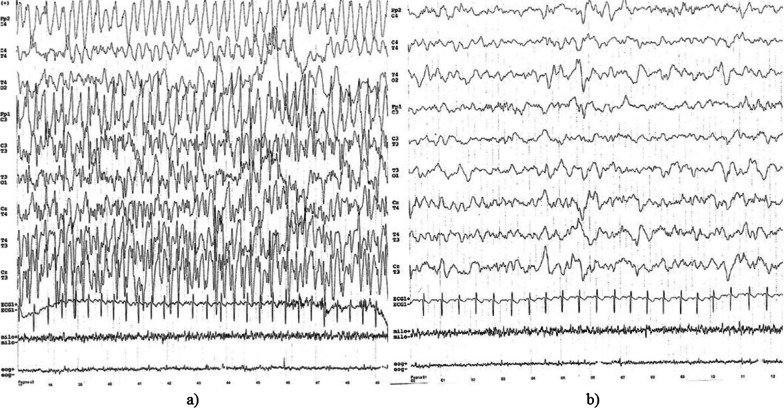
**a** Ictal-EEG. **b** Inter-ictal EEG

L.H. was admitted to the department of Pediatrics at the age of 12-months for the onset of seizure clusters arising during awakening under conditions of apyrexia. The episodes resolved on their own within 1 min and were characterized by loss of consciousness, generalized stiffness and gaze deviation to the right. She also presented prolonged febrile seizures that were treated with benzodiazepines. Family and personal histories were uneventful. At first examination, there were neither physical or facial dysmorphisms nor other comorbidities. The neurological examination was normal and blood laboratory investigations showed no signs of infection. The ictal-EEG showed frontal theta activity of the left hemisphere followed by sharp waves on the same derivations. Slow waves spread on the frontal derivations of the right hemisphere, followed by diffuse slow waves except on the right posterior derivations (Fig. 1a). Inter-ictal EEG was normal (Fig. 1b). Brain MRI resulted negative for parenchymal or vascular diseases.

**Fig. 2:**
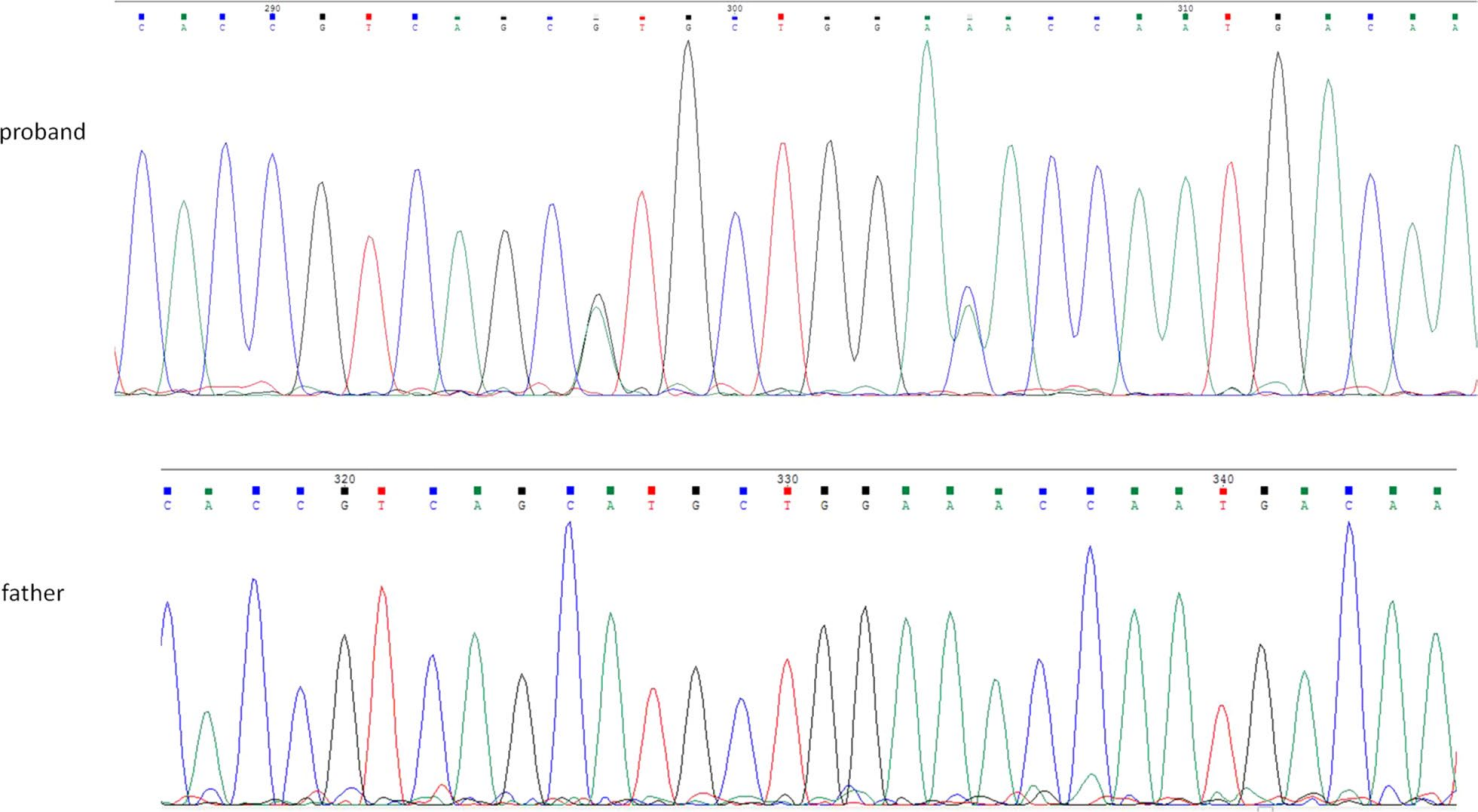
Sanger sequencing confirmed the two variants in the proband and her father

The targeted resequencing analysis of a panel of 40 genes responsible for epilepsy with febrile seizures identified the two variants c.1006G > A (p.Val336Met) and c.1014C > A (p.Asp338Glu) in the gene *PCDH19* (canonical transcript NM_001184880.2). Sanger sequencing confirmed the presence of these variants (Fig. 2). The initial therapy was based on Carbamazepine. However, due to the refractoriness, Clonazepam was added to the therapy. Seizures frequency prior to antiepileptic treatment was more than one episode per day. The double antiepileptic therapy achieved the disappearance of seizures except for sporadic, brief seizures during infectious episodes. Since the age of 2 years and 8 months she is seizure-free and she is currently treated only with Carbamazepine. From 2 years of age EEG controls were persistently normal. Bayley Scales of Infant and Toddler Development–III were normal too. She reached normal score in cognitive, fine and gross motor, social-emotional and adaptive behavior scales. The language score was at the lower limit of the normal range due to a deficiency of the expressive communication. The receptive communication was normal for the age. She attained the expected developmental milestones without any sign of cognitive and behavior disorders.

## Discussion and conclusions

**Fig. 3:**
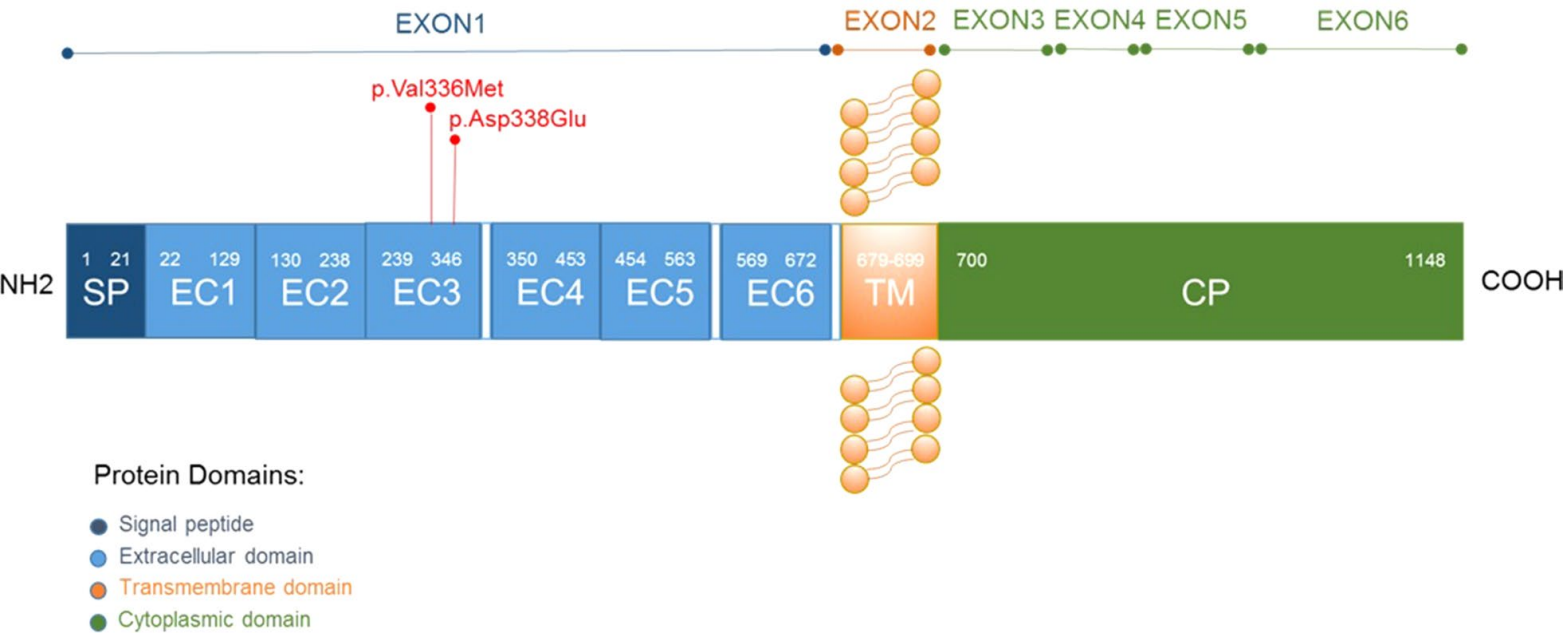
A schematic representation of exons of the PCDH19 gene is depicted at the top of the figure. The protein domains encoded by the corresponding exons are illustrated below. In red are underlined the two missense variants identified in our patient (p.Val336Met and p.Asp338Glu), both localized in the third extracellular (EC) domain. TM: transmembrane domain; CP: cytoplasmic domain; SP: signal peptide

We report a case of infantile seizure presenting as FS + caused by new varaints of the *PCDH19* gene. The clinical suspicion of PCDH19-related epilepsy that prompted us to perform genetic investigations was the presence of seizure clusters with early onset. In addition, FS + especially when associated with family history of epilepsy is highly suspicious for genetic forms of epilepsy, though the indication of genetic testing is under debate. The absence of positive familiar history of our patient could mislead from genetic epilepsy, however, x-linked genomic variants can occur in asymptomatic males and the percentage of de novo genomic variants is not negligible. Our case was associated with the two new variants c.1006G > A (p.Val336Met) and c.1014C > A (p.Asp338Glu) located in *cis* in the exon 1 of the *PCDH19* gene as depicted in Fig. 3. Both variants were inherited from the healthy father. The missense variations that were identified are not reported in the literature, in fact they are absent from population database (GnomAD, ExAC, 1000G).They both lie in highly conserved amino acid residues within the third extracellular domain. Both missense variants are predicted to be pathogenic by 10 different prediction tools such as MutationTaster, Mutation Assessor, SIFT, Polyphen, CADD, LRT, MutPred, List-S2, PROVEAN, M-CAP. These results confirm previous observation about the distribution of missense variants in different extracellular domains among epileptic patients, in contrast with healthy population, showing missense variations in other domains (transmembrane or cytoplasmic) of the *PCDH19* protein [10]. In addition, high frequencies of genomic variants were found at two coding sequence positions, 1019 and 1091 of the *PCDH19* gene identifying a mutational *hot spot* within exon 1 for missense variants [10]. Even those observed in our patient, located in positions 1014 and 1006, fell into this region strengthening the thesis of their pathogenic role in the clinical phenotype. Moreover, the presence of two variants in the same allele appears a very rare phenomenon in monogenic disorders. This could be explained by the presence of mutational "hot spot" in this part of exon1, probably related to some external factors, such as the high susceptibility of codons to variants and slow DNA repair rates.

Interestingly, the presence of two variants in the same protein domain does not adversely affect the expression of the disease in our patient. Only five other cases in the literature showed two coexisting genomic variants, all characterized by cognitive impairment or drug resistance. Seizure types associated with *PCDH19* mutation vary from tonic–clonic, tonic, atonic, absences, myoclonic jerks to Encephalopathies Dravet-like and partial seizures [11]. Our patient showed a mild form of epilepsy with left frontal activity. In addition, the absence of behavioral disorders and cognitive impairment could indicate a less severe phenotypic expression of these variants with an apparently better prognosis. Indeed PCDH19-related epilepsy is generally characterized by a poor response to antiepileptic therapy with frequent cognitive and/or psychiatric involvement that dominates the clinical expression in adulthood. Treatment of PCDH19-related epilepsy is limited by drug resistance and by the absence of specific treatment indications. However, familial mutations have the same reactivity to antiepileptic drugs suggesting a correlation between mutation and therapeutic response. Indeed, in two different families, sisters with PCDH19-related epilepsy caused by inherited mutation had the same response to the therapy [12]. According to our experience, the association of Carbamazepine and Clonazepam has led to a good control of epilepsy. Thus, the finding of these variants could indicate a better prognosis of these patients and suggest this therapeutic approach.

Strength of this case report is represented by the detection of two novel variants expanding the spectrum of *PCDH19* genomic variants associated with epilepsy. The description of just a single case poses a limitation. However, we hope with this work to give a contribution in the analysis of genotype–phenotype correlation of this syndrome and to highlight the importance of a complete diagnostic framework in patients presenting FS + , not only for diagnosis but also for prognosis.

## Data Availability

The datasets generated and/or analysed during the current study have been submitted in Genome Variation Map (GVM; https://ngdc.cncb.ac.cn/gvm/, accession number: GVM000339, project number: PRJCA009711).

## Abbreviations

PCDH19: Protocadherin 19
EFMR: Epilepsy and mental retardation limited to female
FS +: Febrile seizure plus
MRI: Brain magnetic resonance imaging
EEG: Electroencephalography
